# A profile of circulating vascular progenitor cells in human neovascular age-related macular degeneration

**DOI:** 10.1371/journal.pone.0229504

**Published:** 2020-02-27

**Authors:** Timothy Catchpole, Timothy D. Nguyen, Alexa Gilfoyle, Karl G. Csaky

**Affiliations:** 1 Retina Foundation of the Southwest, Dallas, Texas, United States of America; 2 Department of Ophthalmology, University of Texas Southwestern Medical Center, Dallas, Texas, United States of America; Boston University School of Medicine, UNITED STATES

## Abstract

**Background/objective:**

A subset of neovascular age-related macular degeneration (nvAMD) subjects appears to be refractory to the effects of anti-VEGF treatment and require frequent intravitreal injections. The vascular phenotype of the choroidal neovascular (CNV) lesions may contribute to the resistance. Animal studies of CNV lesions have shown that cells originating from bone marrow are capable of forming varying cell types in the lesions. This raised the possibility of a similar cell population in human nvAMD subjects.

**Materials and methods:**

Blood draws were obtained from subjects with active nvAMD while patients were receiving standard of care anti-VEGF injections. Subjects were classified as refractory or non-refractory to anti-VEGF treatment based on previous number of injections in the preceding 12 months. Peripheral blood mononuclear cells (PBMCs) were isolated and CD34-positive cells purified using magnetic bead sorting. The isolated cells were expanded in StemSpan SFEM media to increase cell numbers. After expansion, the cells were split and plated in either endothelial or mesenchymal promoting conditions. Phenotype analysis was performed via qPCR.

**Results:**

There was no significant difference in the number of PBMCs and CD34-positive cells between refractory and non-refractory nvAMD subjects. The growth pattern distribution between endothelial and mesenchymal media conditions were very similar between refractory and non-refractory subjects. qPCR and immunostaining demonstrated positive expression of endothelial markers in endothelial media, and markers such as NG2 and αSMA in mesenchymal media. However, analysis of subsequent samples from AMD subjects demonstrated high variability in both the numbers and differentiation properties of this cell population.

**Conclusions:**

CD34+ cells can be isolated from nvAMD subjects and show both endothelial and pericyte-like characteristics after differentiation in certain media conditions. However, nvAMD subjects show high variability in both numbers of cells and differentiation characteristics in repeat sampling. This variability highlights the importance of taking multiple samples from nvAMD subjects for any clinical trials focused on biomarkers for the disease.

## Introduction

The estimated prevalence of age-related macular degeneration (AMD) in the US population aged 50 years and older is 6.5%[1]. The most common form is dry AMD characterized by the presence of drusen in the macula. The disease may advance to neovascular AMD (nvAMD) characterized by the growth of aberrant blood vessels under the retina termed choroidal neovascularization (CNV). nvAMD accounts for 10–15% of cases of AMD, but is responsible for more than 80% of severe vision loss and legal blindness attributable to AMD[2]. However, the exact pathogenic mechanisms underlying CNV development are still poorly understood.

The primary therapy for nvAMD is intravitreal injections of anti-VEGF agents. While this approach has been shown to improve visual acuity in roughly 1/3 of patients, the number of injections to attain this outcome can vary widely. For example, the Comparison of Age-Related Macular Degeneration Treatment Trials (CATT) study demonstrated a mean of 5.6–6.3 injections per year were required to achieve this visual outcome. However, when assessing nvAMD disease activity on a monthly basis in patients undergoing treatment, some subjects required considerably more injections[3, 4]. For purposes of the present study these subjects will be considered refractory to anti-VEGF treatment, while subjects requiring the mean number (or fewer) of injections of anti-VEGF agents to be non-refractory to treatment. The factors responsible for this variable response to treatment remain speculative. Some of the mechanisms proposed include up-regulation of alternative pro-angiogenic signaling pathways or the development of anti-therapeutic antibodies targeting the anti-VEGF agents[5, 6]. Genetic linkages to treatment response are controversial, with some studies finding links to treatment frequency with alleles in genes including VEGFA[7, 8], while studies with larger sample sizes were unable to replicate this finding[9].

One aspect of the CNV lesion that could influence anti-angiogenic resistance involves the vascular phenotype. A study demonstrated in both animal and human models that blood vessels resistant to anti-VEGF therapy were supported by pericytes and immunoreactive for markers such as NG2, PDGFRβ and αSMA[10]. It has been demonstrated in an animal model of CNV that circulating bone marrow derived cells migrate into and may contribute to the developing vascular complex forming both endothelial and pericyte cells[11]. Isolated circulating CD34+ cells have been shown to be capable of forming both endothelial and pericyte-like cells in-vitro dependent on culture conditions[12]. Based on these pluripotent aspects, circulating CD34+ cells can be termed vascular progenitor cells (VPrCs). Based on the hypothesis that circulating CD34+ cells may contribute to CNV pathogenesis, these differentiation pathways that the CD34+ cells take may affect the growth and cellular composition of the CNV lesions, which in turn may be responsible for the treatment response in nvAMD.

The circulating cell population in nvAMD subjects has found increased relevancy in recent years. Hypomethylation of the *IL-17RC* promoter was reported in the peripheral blood mononuclear cell (PMBC) population in subjects with both dry and wet forms of AMD. This was associated with an increased frequency of CD14^+^IL-17RC^+^ monocytes in the peripheral blood of AMD patients[13]. The same report found increased expression of IL-17RC in macular tissues of AMD subjects, demonstrating that epigenetic changes in circulating cells can have an effect on retinal tissues involved with nvAMD.

In the present study, we attempted to isolate the CD34+ population from human nvAMD subjects, and to examine the proliferative and differentiation characteristics of these cells. In follow-up we then sought to determine if different characteristics of these cells are associated with refractory treatment response to intravitreal injections in nvAMD subjects.

## Materials and methods

### Subject recruitment

A total of 102 subjects were recruited for the study. 42 subjects were classified as refractory and 60 classified as non-refractory nvAMD. Demographic data is outlined in **Table 1**. Subjects were classified as refractory or non-refractory to anti-VEGF treatment based on frequency of anti-VEGF injections in the preceding 12 months prior to recruitment. Refractory subjects had received 7 or more anti-VEGF injections in the preceding 12 months. Non-refractory subjects had received 6 or less anti-VEGF injections in the preceding 12 months. These numbers were based on recruiting nvAMD subjects that were above or below the average number of anti-VEGF injections/y as determined by the CATT studies[3, 4]. The rationale for re-injections was determined by the individual treating physicians and was based on status of retinal fluid on optical coherence tomography (OCT), vision change or presence of retinal hemorrhage. All subjects received ranibizumab (Lucentis^®^), bevacizumab (Avastin^®^) or aflibercept (Eylea^®^) injections in their treatment history. Written informed consent was obtained from each subject before any blood draws were performed. The research adhered to the tenets of the Declaration of Helsinki. The research protocol was approved by the Institutional Review Board of Aspire IRB (Santee, CA 92071).

**Table 1:** Demographic data.

Group	Age	Baseline VA (logMar)	Gender		Smoking status	
	Mean (SD)	Mean (SD)	F (n)	M (n)	Non-Smoker (n)	Current/previous smoker (n)
Refractory (n = 42)	77.45 (7.6)	0.38 (0.28)	23	19	38	4
Non-refractory (n = 60)	80.77 (6.7)	0.33 (0.26)	31	29	52	8

### Peripheral blood mononuclear cell (PBMC) isolation

An initial blood draw of 32 milliliters (mL) was obtained from each subject. A second blood draw was obtained 1–12 months after the first from 83 of the 102 subjects. PBMCs were isolated with Vacutainer CPT tubes (Becton Dickinson, New Jersey, USA) following manufacturer’s instructions. The number of PBMCs isolated was counted using a Countess automated cell counter (Invitrogen, Massachusetts, USA).

### CD34+ cell sorting

The isolated PBMCs were suspended in bead sorting buffer (PBS, 2mM EDTA, 0.5% BSA) at a concentration of 0.5 x 10^8^ cell/300μl. 100μL of FcR Blocking reagent/10^8^ cells and 100μl of CD34 microbeads/10^8^ cells (Miltenyi Biotec, Bergisch Gladbach, Germany) were added to the cell suspension. The cells were incubated for 30 mins at 4°C, and then washed with 5mL of bead buffer. The cells were run through two MS columns (Miltenyi Biotec) according to manufacturer’s instructions. Cell numbers in the positive and negative fractions were determined using the Countess cell counter.

### Flow cytometry

Cells (5 x 10^6^) from the unsorted PBMC suspension, CD34 positive and negative fractions were suspended in 100μL of bead sorting buffer. 10 μL of CD34-FITC antibody (Miltenyi-Biotec) was added to the cell suspensions. The samples were incubated for 10 mins at 4°C in the dark. 500μL of bead sorting buffer was added to wash the cells. The labeled samples were spun down and resuspended in 500μL buffer. 125μl of 4% Paraformaldehyde was added and the cells were fixed for 10 mins at 4°C. After fixation, the cells were spun down and resuspended in 500μL buffer for analysis. The cell sorting was performed on a FACScan instrument. FlowJo v8.8.6 was used to analyze the results.

### Expansion of CD34+ cells

CD34+ cells were cultured at a concentration of 5 x 10^4^ cells/500μl/2cm^2^ in serum free StemSpan Medium (StemCell Technologies, Vancouver, Canada) for seven days. The media was supplemented with StemSpan CC-100 (StemCell Technologies), which contains Flt-3L, SCF, IL-3 and IL-6.

### Differentiation of CD34+ cells

After CD34+ cell population expansion, the cells were split into two halves and plated in Endothelial Cell Growth Medium MV2 and Pericyte Growth Medium (Promocell, Heidelberg, Germany) at a concentration of 1 x 10^6^ cells/2ml/9.6cm^2^ in fibronectin-coated tissue culture dishes.

### Quantitative PCR analysis

Total RNA was isolated from the differentiated cell populations using the *mir*Vana Isolation Kit (Ambion, Massachusetts, USA) following manufacturer’s instructions. cDNA was produced using the High Capacity cDNA Reverse Transcription Kit (Applied Biosystems, Massachusetts, USA). For TaqMan assays, 10ng of total RNA was used for each reaction. Assays included Von Willebrand Factor (vWF; Applied Biosystems assay ID Hs00169795_m1), Neuron-Glial antigen 2 (NG2, Applied Biosystems assay ID Hs00361541_g1), Collagen type 1 alpha 1 (COL1A1, Applied Biosystems assay ID Hs00164004_m1) and 18S (Applied Biosystems assay ID Hs99999901_s1). Assays were performed in a 96-well optical plate in a 7300 Real Time PCR System (Applied Biosystems), following protocols supplied by the manufacturer. PCR conditions for the assays were 50°C for 2mins, followed by 95°C for 10 mins, followed by 40 cycles of 95°C for 15secs and 60°C for 60secs. Assays were carried out in triplicate, and the 18S assay was utilized as the endogenous control for the samples. Expression analysis was performed using RQ Manager Version 1.2.1 (Applied Biosystems).

### Immunostaining

After expansion, CD34+ cells were plated on an 8-well slide in endothelial and pericyte growth medium. For immunostaining, the cells were washed with 3 cycles of PBS, and then fixed with 4% Paraformaldehyde at 4°C for 30 mins. After fixation the cells were blocked in PBST (0.2% Triton X-100) + 1% BSA for 60 mins. Primary antibodies (anti-VWF #A0082, Dako Agilent, Santa Clara, USA; anti-SMA #M0851, Dako; anti-COL1A1 #ABT257, MilliporeSigma, Massachusetts, USA) were diluted 1/100 in PBST + 1% BSA and added as appropriate overnight at 4°C. Cells were washed x3 with PBS and secondary antibodies (Goat anti-Rabbit IgG Alexa Fluor-555 #ab150074, Abcam, Cambridge, USA; Goat anti-mouse IgG Alexa Fluor-488 #ab150105, Abcam) were added for 4 hours at RT. Cells were washed x3 with PBS and nuclei were stained with Hoechst 33342 (Tocris, Bristol, UK) for 10mins. Coverslips were mounted with Prolong Diamond Mountant (Invitrogen). Imaging was performed on a LSM 800 confocal microscope (Zeiss, Oberkochen, Germany). Negative controls were obtained by following the above procedure without the addition of primary antibodies. Images of the negative controls were obtained using identical laser settings and imaging conditions on the confocal microscope.

### Statistical analysis

Statistical analysis was performed in GraphPad Prism Version 6.0h and JASP 0.11.1. For direct comparisons between two conditions, student’s *t*-tests were used to determine significant differences, as described in the results section. Analysis of Table 2 was performed with a chi test of independence. A *P* value of ≤0.05 was considered significant.

**Table 2:** Differentiation of CD34+ cells in first blood draw.

Classification	No of Subjects (n)	CD34+ growth and differentiation			
		Growth in both EGM-MV2 and Pericyte media	Growth in EGM-MV2 media	Growth in Pericyte media	No growth observed in both media conditions
Refractory	42	6 (14.28%)	4 (9.52%)	16 (38.10%)	16 (38.10%)
Non-Refractory	60	13 (21.66%)	4 (6.67%)	18 (30.00%)	25 (41.67%)

## Results

### Isolation of CD34+ cells in nvAMD subjects

**Fig 1:**
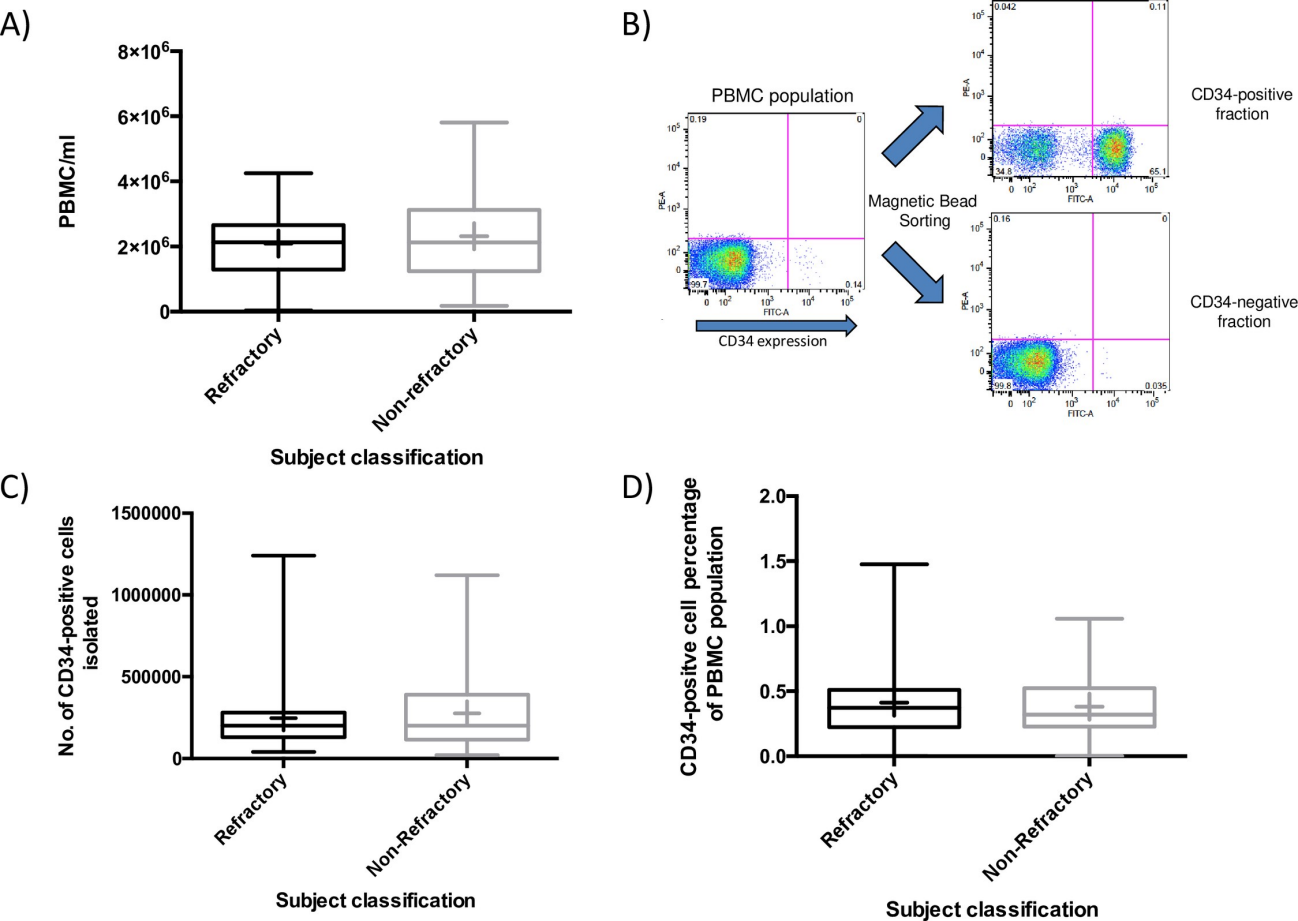
Sorting of CD34+ cells from PBMC population. **A)** Box and whisker plots comparing the numbers of PBMC per mL between refractory and non-refractory nvAMD subjects. Plots show the maximum value, 3^rd^ quartile, median, 1^st^ quartile and minimum values. Mean shown as +. **B)** FACS analysis of magnetic bead sorting showing percentage of CD34+ cells in unsorted PBMC population, CD34+ fraction and CD34- fraction. **C)** Box and whisker plots comparing the numbers of CD34+ cells isolated between refractory and non-refractory nvAMD subjects. **D)** Box and whisker plots comparing percentage of CD34+ cells in PBMC population between refractory and non-refractory nvAMD subjects.

PBMC’s were isolated from an initial blood draw from each nvAMD subject. We observed an average of 2.092 x 10^6^ ± 9.95 x 10^5^ (mean, SD) PBMC/mL in the refractory group, and an average of 2.315x10^6^ ± 1.39x10^6^ PBMC/mL in the non-refractory group (**Fig 1A**). There was no significant difference in average PBMC numbers between these two groups (*t*-test, P = 0.376). The overall viability for the PBMC population was 94.25 ± 5.7%. There was no statistical difference in viability between refractory and non-refractory subjects (94.14 ± 5.84% vs 94.33 ± 5.65% respectively; *t*-test, P = 0.869). To analyze the effectiveness of the magnetic bead isolation of the CD34+ cell population from the PBMC population, we performed Flow Cytometry (FACS) analysis for CD34 staining. **Fig 1B** illustrates the results of a sorting experiment. The unsorted PBMC population contained 0.14% CD34+ cells (lower-right quadrant of FACS image). After magnetic bead selection, the collected fraction had 65.1% CD34+ cells. The percentage of CD34+ cells in the uncollected fraction dropped to 0.035%.

The numbers of CD34+ cells isolated from nvAMD subjects were highly variable, ranging from 2x10^4^ to 1.24x10^6^ cells. The average number of CD34+ cells isolated from the refractory group was 2.473x10^5^ ± 2.09x10^5^, while the average number of CD34+ cells in the non-refractory group was 2.762x10^5^ ± 2.33x10^5^ (**Fig 1C**). There was no significant difference in CD34+ cell numbers between the two groups (*t*-test, P = 0.528). CD34+ cells comprised an average of 0.412% ± 0.26% of the PBMC population in the refractory nvAMD group (**Fig 1D**). In the non-refractory group, CD34+ cells comprised an average of 0.381% ± 0.22% of the PBMC population. Again, there was no significant difference between the two groups (*t*-test, P = 0.515). The overall viability of the isolated CD34 population was 73.66 ± 15.27%, with no difference between the viability observed in refractory and non- refractory subjects (71.98 ± 14.59 vs 74.83 ±15.75; *t*-test, P = 0.366).

### Expansion of CD34+ cells

**Fig 2:**
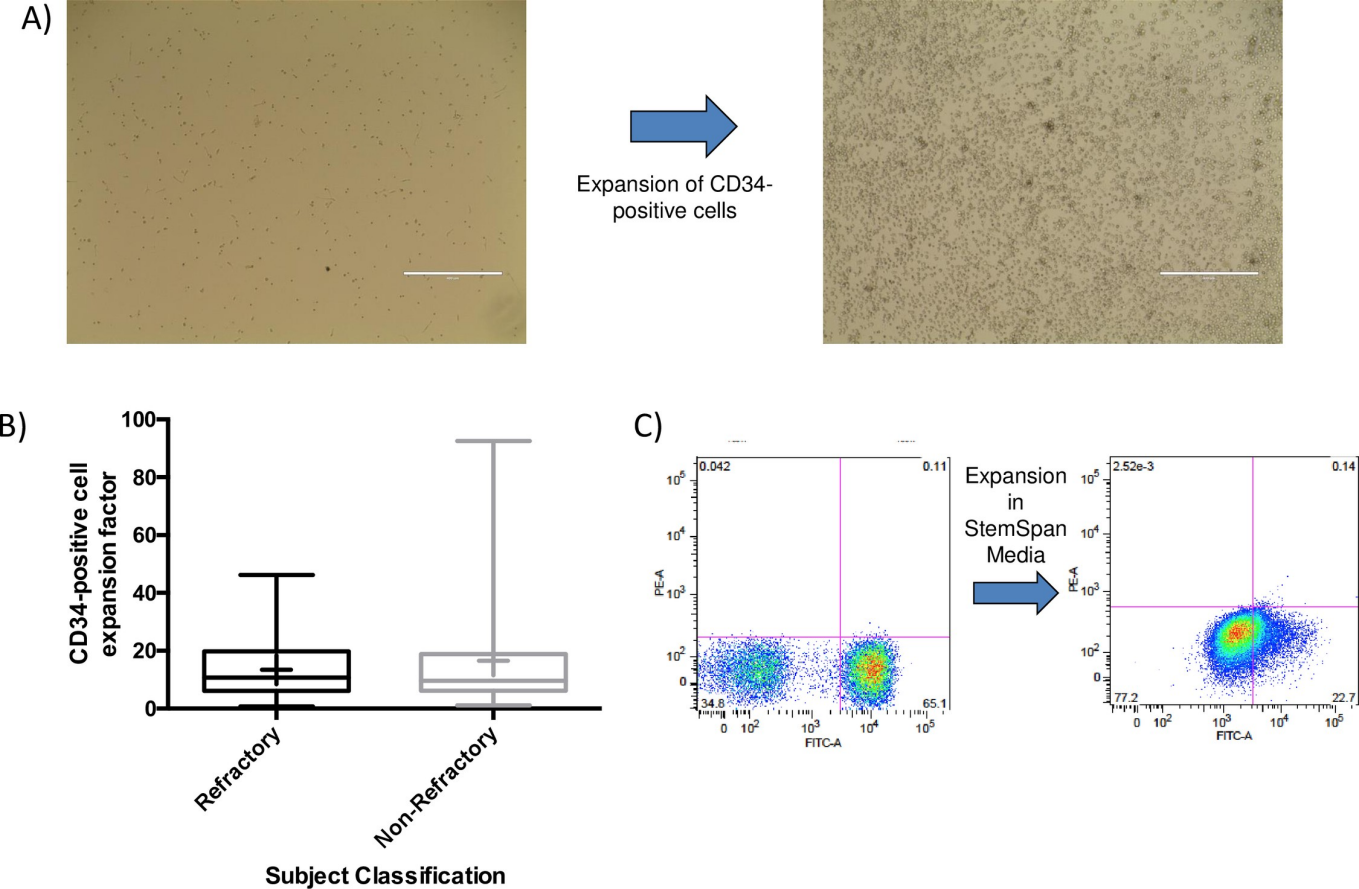
Expansion of CD34+ cells **A)** Images of isolated CD34+ cells 1 day (left image) and 7 days (right image) after plating in StemSpan media. **B)** Box and whisker plots comparing the expansion factor of CD34+ cells between refractory and non-refractory nvAMD subjects. **C)** FACS analysis of CD34 expression in a CD34+ population before and after expansion in StemSpan media.

To increase the number of CD34+ cells available for further experiments, we expanded the CD34+ cell population in StemSpan SFEM media + CC100 supplement (StemCell Technologies) over a period of 7 days. **Fig 2A** shows the growth of this population over this time period. There was little sign of cell attachment over the expansion period, and the cells remained in a circular expansion state. The expansion factor (number of cells counted at end of expansion period/Number of cells at beginning of expansion period) was highly variable between the CD34+ population isolated from nvAMD subjects (**Fig 2B**), ranging from a factor of 0.81 to 92.57. There was no significant difference in expansion factors between the refractory and non-refractory groups (*t*-test, P = 0.341; 13.44 ± 9.89 for refractory vs 16.51 ± 18.78 for non-refractory). The viability of the expanded CD34+ population (82.32 ± 14.73%) rose slightly compared to pre-expansion levels, again with no difference between the viability observed in refractory and non- refractory subjects (82.09 ± 12.56 vs 82.48 ± 16.13%; *t*-test, P = 0.898).

To determine if expansion in StemSpan media was affecting CD34 expression, we performed FACS analysis on the expanded population (**Fig 2C**). The percentage of cells expressing CD34 dropped from 65.1% in the pre-expanded state to 22.7% after 7 days of expansion.

### Differentiation of CD34+ cells under endothelial and mesenchymal promoting conditions

**Fig 3:**
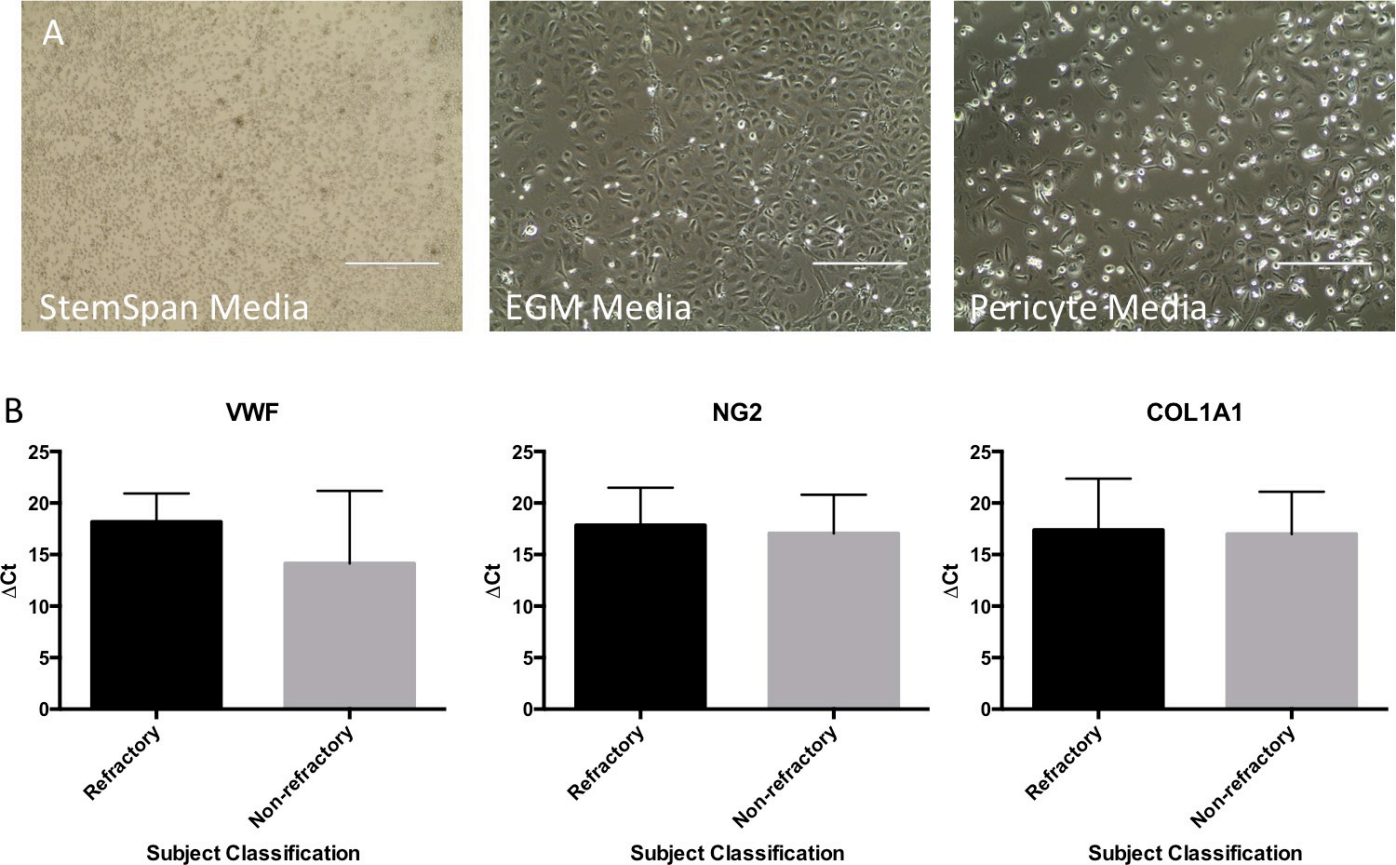
Differentiation of CD34+ cells. **A)** Images of isolated CD34+ cells after expansion in StemSpan media (left image), culturing in EGM media (center image) and culturing in pericyte media (right image). **B)** Comparison of ΔC_T_ values of VWF, NG2 and COL1A1 (endogenous control 18S) between refractory and non-refractory subjects.

To examine the differentiation of the isolated CD34+ populations, the cells were plated in endothelial-promoting conditions (EGM-MV2 media, Promocell) and separately in mesenchymal-promoting conditions (Pericyte Growth Media, Promocell). Cells were cultured over 3–4 weeks and monitored for attachment and growth (**Fig 3A**). The growth patterns of CD34+ cells from nvAMD subjects are shown in Table 2. While a large number of subjects did not demonstrate CD34+ growth in either media condition, other subjects showed growth in either EGM-MV2 or pericyte media, while a small proportion of subjects demonstrated positive cell growth in both media conditions. A chi test of independence was performed to examine the relationship between response to anti-VEGF treatment (refractory or non-refractory) and their cells differentiation pattern in separate media conditions. The relationship between these variables was not significant (X^2^(3, N = 102) = 1.544, P = 0.672), suggesting that refractoriness to anti-VEGF treatment does not influence CD34+ cell response to different media conditions.

To confirm the phenotype of cells derived in the separate media conditions, the expression of Von-Willebrand Factor (VWF, endothelial cell marker) and Chondroitin Sulfate Proteoglycan 4/NG2 (CSPG4, pericyte marker) was analyzed via qPCR in the cells grown in the separate media conditions in subjects that showed successful cell growth in both differentiation conditions. Expression of VWF in cells derived in endothelial media had an average RQ value of 1405.21 when compared to cells derived in pericyte media from the same subject. Conversely, expression of NG2 in cells grown in the pericyte media had an average RQ value of 31.74 when compared to cells derived in endothelial media. To test whether the pericyte media-derived cells can contribute to the fibrotic response in nvAMD, we tested the cells for expression of COL1A1, a component of Collagen 1. Expression of COL1A1 in cells derived in pericyte-media had an average RQ value of 172.21 when compared to cells derived in endothelial media. To confirm expression of endothelial and mesenchymal markers, cells were immunostained for VWF and αSMA (**S1 Fig**). Expression of COL1A1 was confirmed in a co-labeling immunostain with αSMA and COL1A1 antibodies (**S2 Fig**).

The ΔC_T_ values for these genes (VWF, NG2 and COL1A1) were compared between refractory and non-refractory subjects to determine if there was a link between expression levels of these genes in the derived cells and response to anti-VEGF treatment (**Fig 3B**). There were no significant differences observed in the expression levels of these genes when comparing refractory and non-refractory subjects.

### Variability of results from intrapatient samples

To examine the variability of the results obtained from the nvAMD subjects, a second blood draw was taken from 83 of the subjects 1–12 months after the first. The numbers of PBMC and CD34+ cells isolated were similar to the first draws, and again there was no significant difference between the refractory and non-refractory subjects in terms of cell numbers and expansion factors of CD34+ cells (**S3 Fig**).

**Fig 4:**
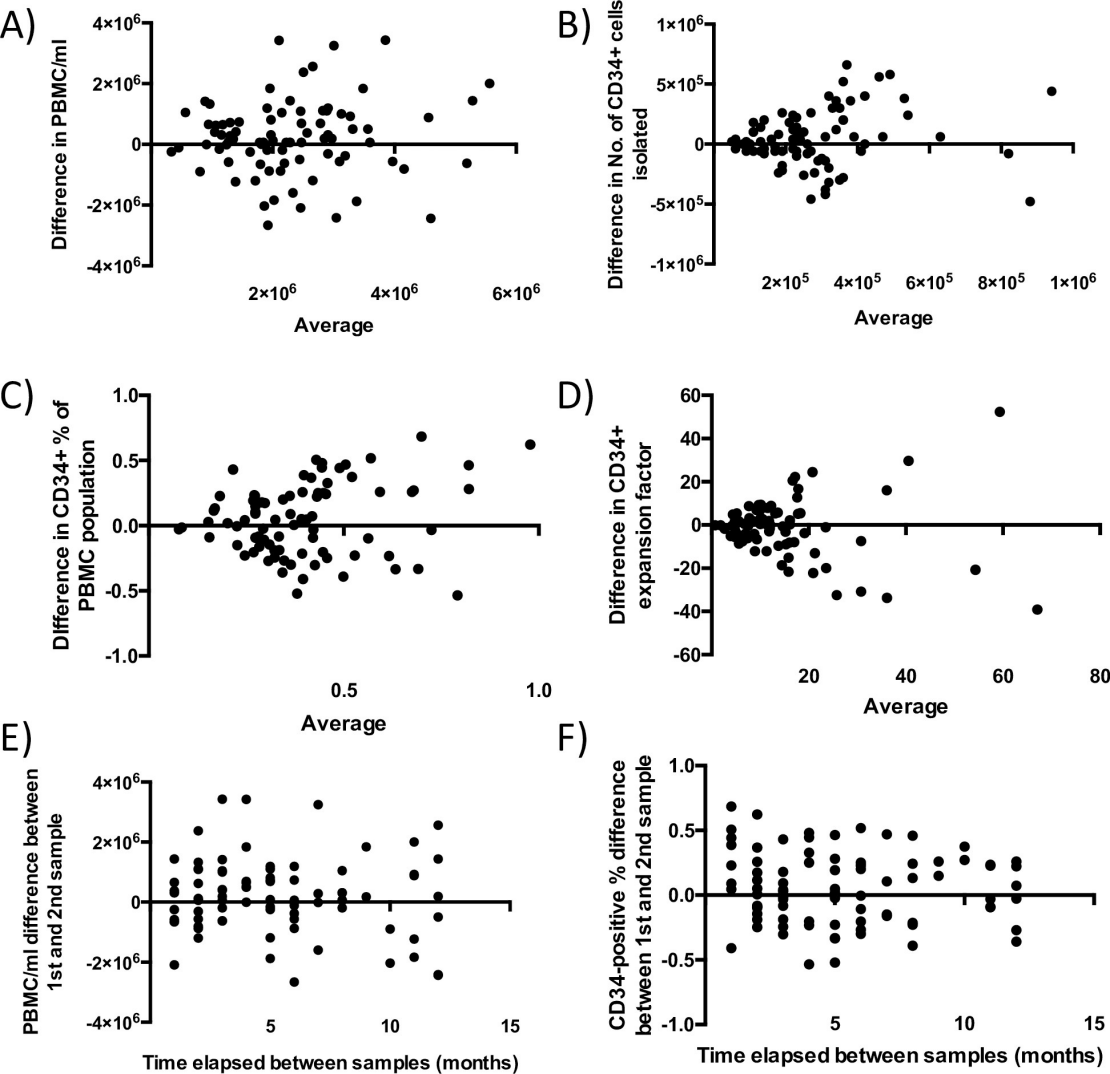
Variability of blood draws. **A)** Bland-Altman analysis of the mean PBMC/ml for each subject vs difference in PBMC/ml between 1^st^ and 2^nd^ blood draw. **B)** Bland-Altman analysis of the mean no. of CD34+ cells isolated for each subject vs difference in no. of CD34+ cells isolated between 1^st^ and 2^nd^ blood draw. **C)** Bland-Altman analysis of the CD34% of PBMC population for each subject vs difference in CD34% between 1^st^ and 2^nd^ blood draw. **D)** Bland-Altman analysis of the CD34+ expansion factor for each subject vs difference in CD34 expansion factor between 1^st^ and 2^nd^ blood draw. **E)** Scatter plot of the difference in PBMC/ml obtained in intra-patient samples vs time elapsed between samples. **F)** Scatter plot of the difference in CD34-positive percentage of the PBMC population obtained in intra-patient samples vs time elapsed between samples.

Test-retest variability was examined for these factors by performing Bland-Altman analyses on the cell numbers obtained (**Fig 4**). For the numbers of PBMC/ml isolated, the bias (mean) was 2 x 10^5^, with 95% Limits of Agreement (LOA) were from -2.2 x 10^6^ to 2.6 x 10^6^ (**Fig 4A**). For the numbers of CD34+ cells isolated, the bias was 4.2 x 10^4^, with 95% LOA from -4.2 x 10^5^ to 5 x 10^5^ (**Fig 4B**). The percentage of CD34+ cells had a bias of 0.05%, with 95% LOA from -0.49 to 0.59 (**Fig 4C**). The CD34+ expansion factor had a bias of -1.43, with 95% LOA from -28.33 to 25.47 (**Fig 4D**). For many subjects, the variability between blood draws was high, with values changing by as much as 100% between draws for the data points analyzed. The time elapsed between samples from the same patient was not linked to an increase in observed variability for both PBMC numbers and CD34-positive percentages (**Fig 4E and 4F**).

**Fig 5:**
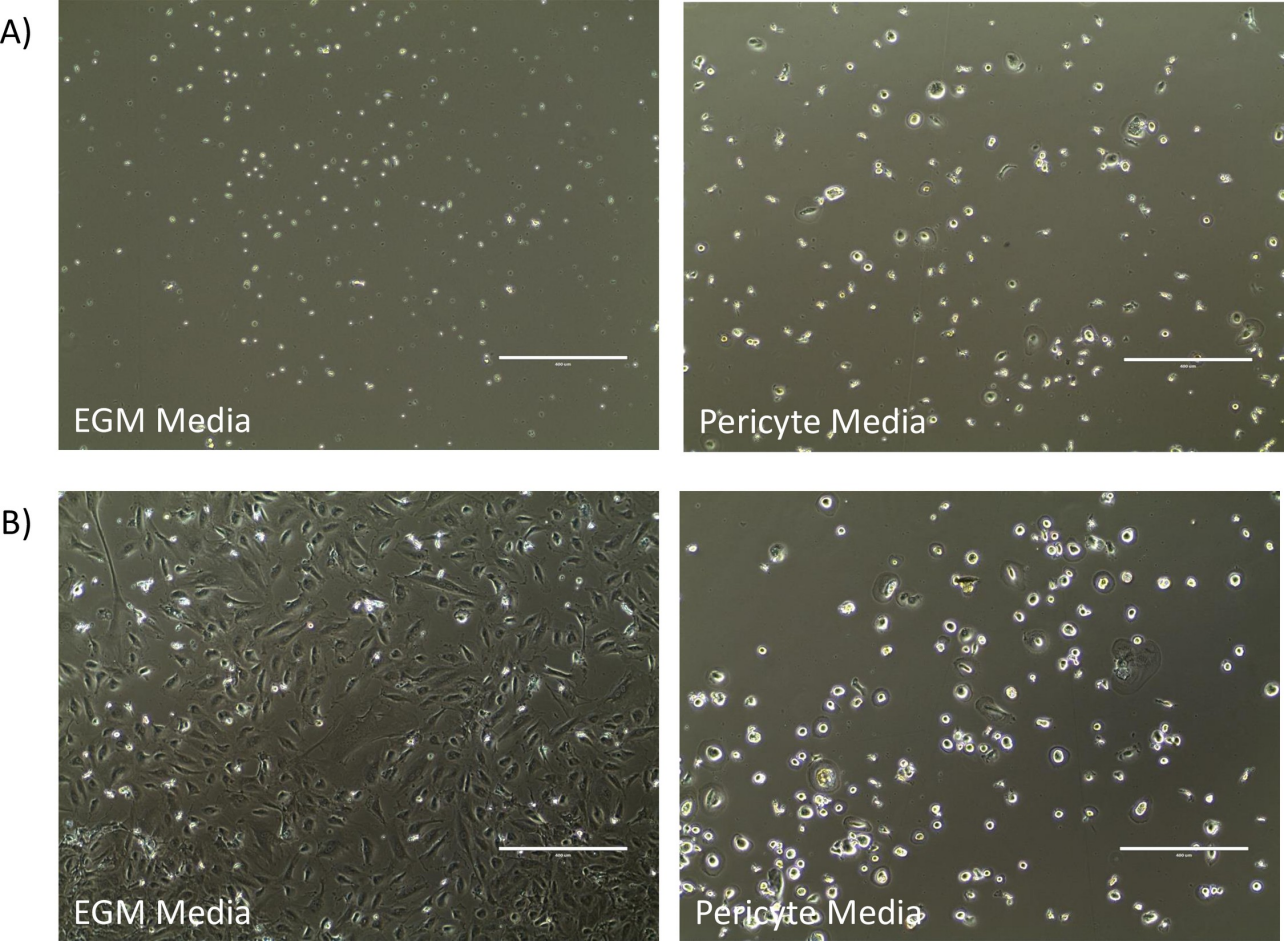
Repeatability of CD34+ differentiation. Comparison of CD34+ cells isolated from a single subject examining differentiation in EGM and Pericyte media from the first blood draw **(A)** vs the second blood draw **(B)**.

As well as variation in the numbers of cells isolated and their expansion properties, there was also variation in the differentiation of the cells. From the 83 redraws obtained, only 30 subjects (36.1%) showed the same CD34+ differentiation patterns as obtained in the first draws. **Fig 5** shows an example of the changing differentiation patterns with results of two draws from a single subject, where there was no cell attachment seen in EGM media in the first draw, and considerable cell attachment and growth seen in EGM media in the second draw.

## Discussion

The present study was designed to test whether circulating VPrC type CD34+ cells exist in patients with nvAMD and whether a link could be established between the properties of the circulating CD34+ cell population in nvAMD subjects and response to anti-VEGF therapy. This is the first study to establish that a population of CD34+ cells can be isolated from elderly patients with neovascular AMD that can be expanded in-vitro and are able to subsequently differentiate into either VWF + endothelial cells or NG2+ pericyte cells under VEGF or PDGF exposure respectively. When comparing the distribution or comparative ratios of these subsequent vascular cells there was no correlation between the frequency of anti-VEGF injections needed and the number of CD34+ cells isolated, their expansion potential and their differentiation characteristics. The primary difficulty encountered in this study is the variability of results gathered from the nvAMD subjects. While some subjects were consistent in terms of cell numbers and differentiation characteristics, a large number of these subjects showed different results between blood draws. The high variability of these cells isolated in our study highlights the importance of taking multiple samples from nvAMD subjects for any clinical trials focused on biomarkers for the disease.

These results are in contrast with our previous study, which examined the expression of Prokineticin-2 (Bv8) in the circulating CD11b+ cell population in nvAMD subjects. In that study low variation from samples taken at points from the same subject, and a significant level of differences between inter-individual samples was found[14]. The major difference between these two studies is that CD11b+ cells make up approximately 25% of the PBMC population, and are relatively straightforward to purify to a highly pure population using magnetic bead sorting (89.6%)[14]. By contrast, CD34+ cells are estimated to comprise as low as 0.2% of the PBMC population using flow cytometry quantification techniques[15]. In the present study it was far more difficult to obtain a pure population of CD34+ cells using magnetic bead sorting (65.1%). Using flow cytometry to purify CD34+ cells may increase the purity of the isolated population, but it increases the difficulty of keeping the cell population sterile for culturing purposes and can alter the state of the sorted cells[16].

The other potential source of variation between tests in this study could be due to the nature of the CD34+ cells themselves. Circulating CD34+ cells arise from hemangioblasts in the bone marrow[17]. Any biological activity that affected the bone marrow population of hemangioblasts would have significant effects on the circulating CD34+ population. For example, smoking, a known risk factor for the development and progression of AMD, has been demonstrated to have a negative effect on cell populations within the bone marrow[18].

Another factor to consider is the heterogeneity of the CD34+ population. While CD34 is expressed on circulating endothelial progenitors, it is also expressed on epithelial progenitors, multipotent mesenchymal stromal cells, and other cell types such as muscle satellite and interstitial cells[19]. While not all these cell types are found in peripheral blood, the methods described in this paper to isolate the cells do not distinguish between the various CD34+ types. It is possible that blood samples from different subjects will express different proportions of these varying types of CD34+ cells, which could contribute to variability.

While this study focused on the phenotypic characteristics of the CD34+ cells, the functional changes of the cells post-differentiation may be an important aspect to consider. As well as their roles as vascular cells, the differentiated cells may also influence surrounding cells in the retina though paracrine actions. Future experiments will include examining cytokine and other signaling molecules in the conditioned media from the differentiated cells.

nvAMD is a very unpredictable disease, both in terms of progression and response to treatment. A biomarker that could provide some indication of these would be very useful in the clinic. Circulating CD34 cells are an attractive prospect, in that the method of isolation is relatively non-invasive and the cells have the potential to affect the cellular composition of the AMD lesions, directly impacting the response to treatment. However, the variability seen in our results means an abundance of caution must be recognized in treating these cells as a biomarker. Any future studies must take the present results into account and be able to demonstrate intrapatient replication in multiple samples from nvAMD patients before a biomarker can be considered clinically relevant.

## Supporting information

S1 Fig Immunostain of endothelial and mesenchymal markers in differentiated cells. **A)** Immunofluorescent (IF) image of CD34+ cells differentiated in endothelial media labeled for VWF (red) and Hoechst (blue). **B)** IF image of CD34+ cells differentiated in pericyte media labeled for αSMA (green) and Hoechst (blue).
(TIF)

S2 Fig Co-labeling immunostain of COL1A1 and αSMA in differentiated cells. **A)** IF image of CD34+ cells differentiated in pericyte media labeled for αSMA (green), COL1A1 (red) and Hoechst (blue).
(TIF)

S3 Fig Sorting of CD34+ cells from PBMC population from second sample. **A)** Box and whisker plots comparing the numbers of PBMC per mL between refractory and non-refractory nvAMD subjects from second blood draw. **B)** Box and whisker plots comparing the numbers of CD34+ cells isolated between refractory and non-refractory nvAMD subjects from second blood draw. **C)** Box and whisker plots comparing percentage of CD34+ cells in PBMC population between refractory and non-refractory nvAMD subjects from second blood draw. **D)** Box and whisker plots comparing the expansion factor of CD34+ cells between refractory and non-refractory nvAMD subjects from second blood draw.
(TIF)

S1 Data (XLSX)

S2 Data (XLSX)

S3 Data (XLSX)

S4 Data (XLSX)

S5 Data (XLSX)

S6 Data (XLSX)
