# Supplementary figures and images for: A profile of circulating vascular progenitor cells in human neovascular age-related macular degeneration
Source: PLoS One. 2020 Feb 27;15(2):e0229504. doi: 10.1371/journal.pone.0229504 (PMC7046286; doi:10.1371/journal.pone.0229504)

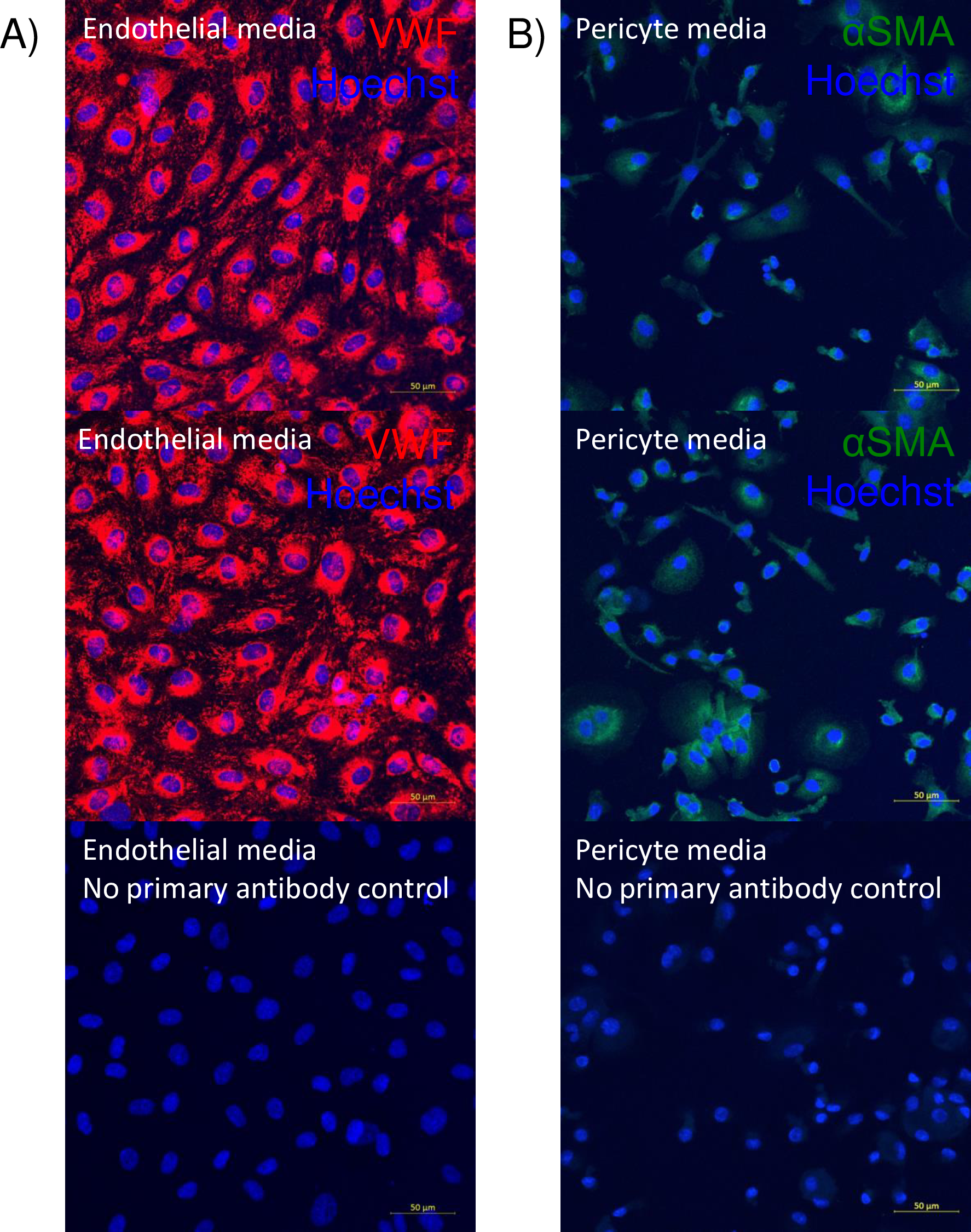

Supplement: S1 Fig — A) Immunofluorescent (IF) image of CD34+ cells differentiated in endothelial media labeled for VWF (red) and Hoechst (blue). B) IF image of CD34+ cells differentiated in pericyte media labeled for αSMA (green) and Hoechst (blue). (TIF) [file pone.0229504.s001.tif]

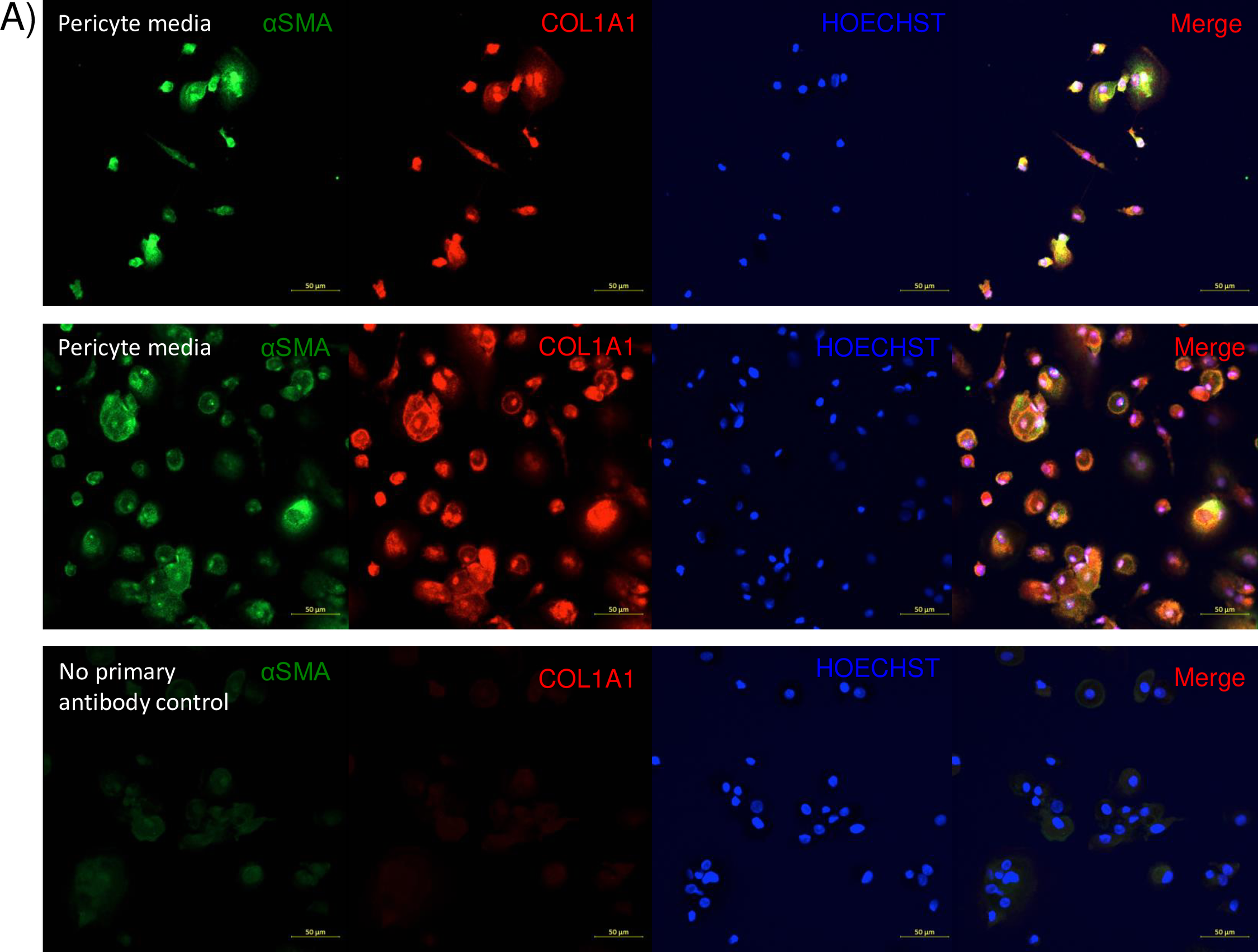

Supplement: S2 Fig — A) IF image of CD34+ cells differentiated in pericyte media labeled for αSMA (green), COL1A1 (red) and Hoechst (blue). (TIF) [file pone.0229504.s002.tif]

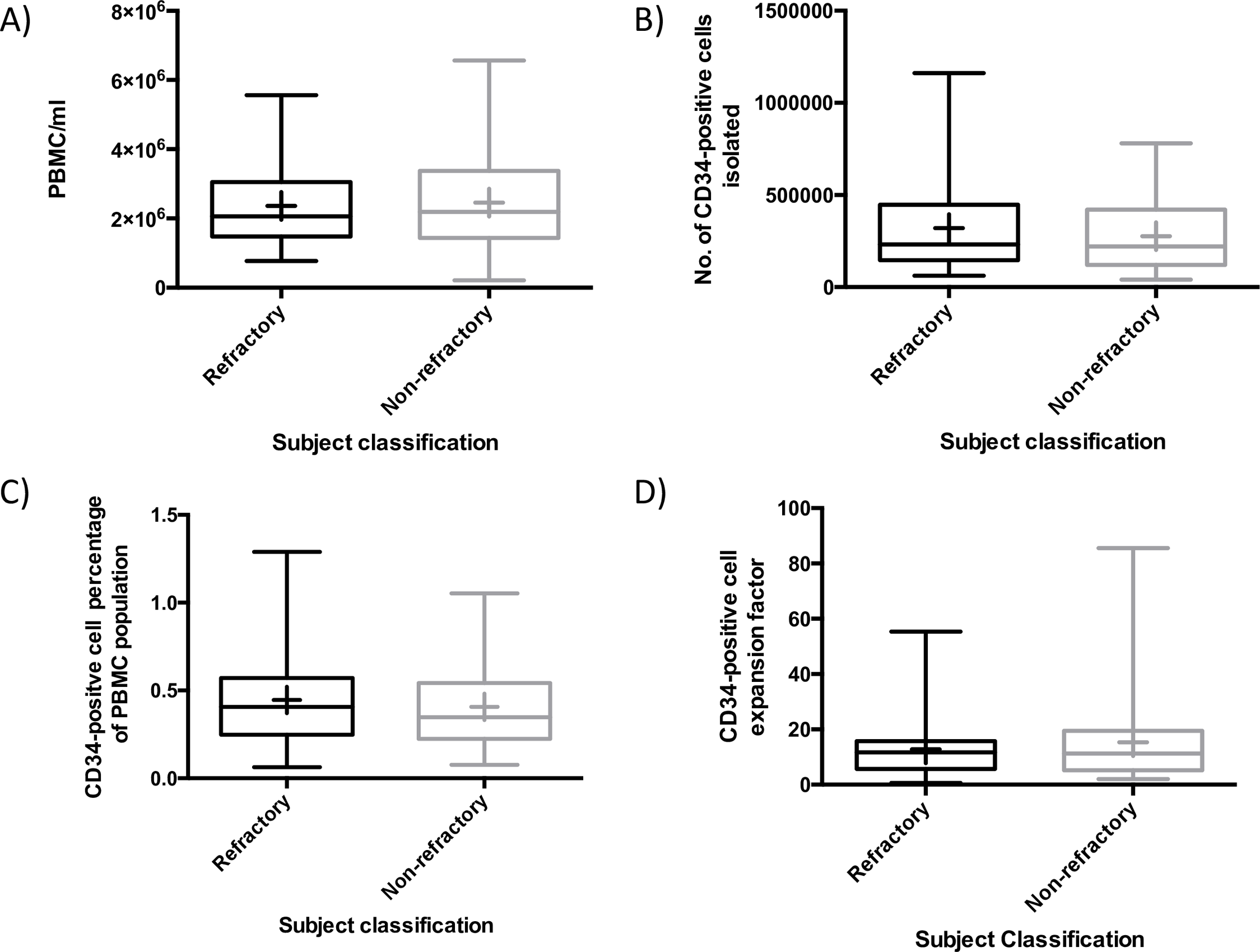

Supplement: S3 Fig — A) Box and whisker plots comparing the numbers of PBMC per mL between refractory and non-refractory nvAMD subjects from second blood draw. B) Box and whisker plots comparing the numbers of CD34+ cells isolated between refractory and non-refractory nvAMD subjects from second blood draw. C) Box and whisker plots comparing percentage of CD34+ cells in PBMC population between refractory and non-refractory nvAMD subjects from second blood draw. D) Box and whisker plots comparing the expansion factor of CD34+ cells between refractory and non-refractory nvAMD subjects from second blood draw. (TIF) [file pone.0229504.s003.tif]
